# Ultrasmall and highly biocompatible carbon dots derived from natural plant with amelioration against acute kidney injury

**DOI:** 10.1186/s12951-023-01795-5

**Published:** 2023-02-23

**Authors:** Xiaoke Wang, Tong Wu, Yingxin Yang, Long Zhou, Shuxian Wang, Jiaxing Liu, Yafang Zhao, Meiling Zhang, Yan Zhao, Huihua Qu, Hui Kong, Yue Zhang

**Affiliations:** 1 Encephalopathy Hospital, The First Affiliated Hospital of Henan University of Chinese Medicine, Zhengzhou, 450000 China; 2 School of Chinese Materia Medica, Beijing University of Chinese Medicine, Beijing, 100029 China; 3 School of Traditional Chinese Medicine, Beijing University of Chinese Medicine, Beijing, 100029 China; 4 Third Affiliated Hospital, Beijing University of Chinese Medicine, Beijing, 100029 China; 5 Key Laboratory of Chinese Internal Medicine of the Ministry of Education, Dongzhimen Hospital Affiliated to Beijing University of Chinese Medicine, Beijing, 100020 China; 6 Center of Scientific Experiment, Beijing University of Chinese Medicine, Beijing, 100029 China; 7 School of Life Science, Beijing University of Chinese Medicine, Beijing, 100029 China

**Keywords:** Carbon dots, Pollen Typhae, Acute kidney injury, Biomass, Rhabdomyolysis

## Abstract

**Background:**

Acute kidney injury (AKI) refers to a tricky clinical disease, known by its high morbidity and mortality, with no real specific medicine for AKI. The carbonization product from *Pollen Typhae* (i.e., Pu-huang in China) has been extensively employed in clinic, and it is capable of relieving the renal damage and other diseases in China since acient times.

**Results:**

Inspired by the carbonization process of Traditional Chinese Medicine (TCM), a novel species of carbon dots derived from *Pollen Typhae* (PT-CDs) was separated and then collected using a one-pot pyrolysis method. The as-prepared PT-CDs (4.85 ± 2.06 nm) with negative charge and abundant oxygenated groups exhibited high solubility, and they were stable in water. Moreover, the rhabdomyolysis (RM)-induced AKI rat model was used, and it was first demonstrated that PT-CDs had significant activity in improving the level of BUN and CRE, urine volume and kidney index, and histopathological morphology in RM-induced AKI rats. It is noteworthy that interventions of PT-CDs significantly reduced degree of inflammatory reaction and oxidative stress, which may be correlated with the basial potential mechanism of anti-AKI activities. Furthermore, cytotoxicity assay and biosafety evaluation exhibited high biocompatibility of PT-CDs.

**Conclusion:**

This study offers a novel relieving strategy for AKI based on PT-CDs and suggests its potential to be a related candidate for clinical applications.

**Graphical Abstract:**

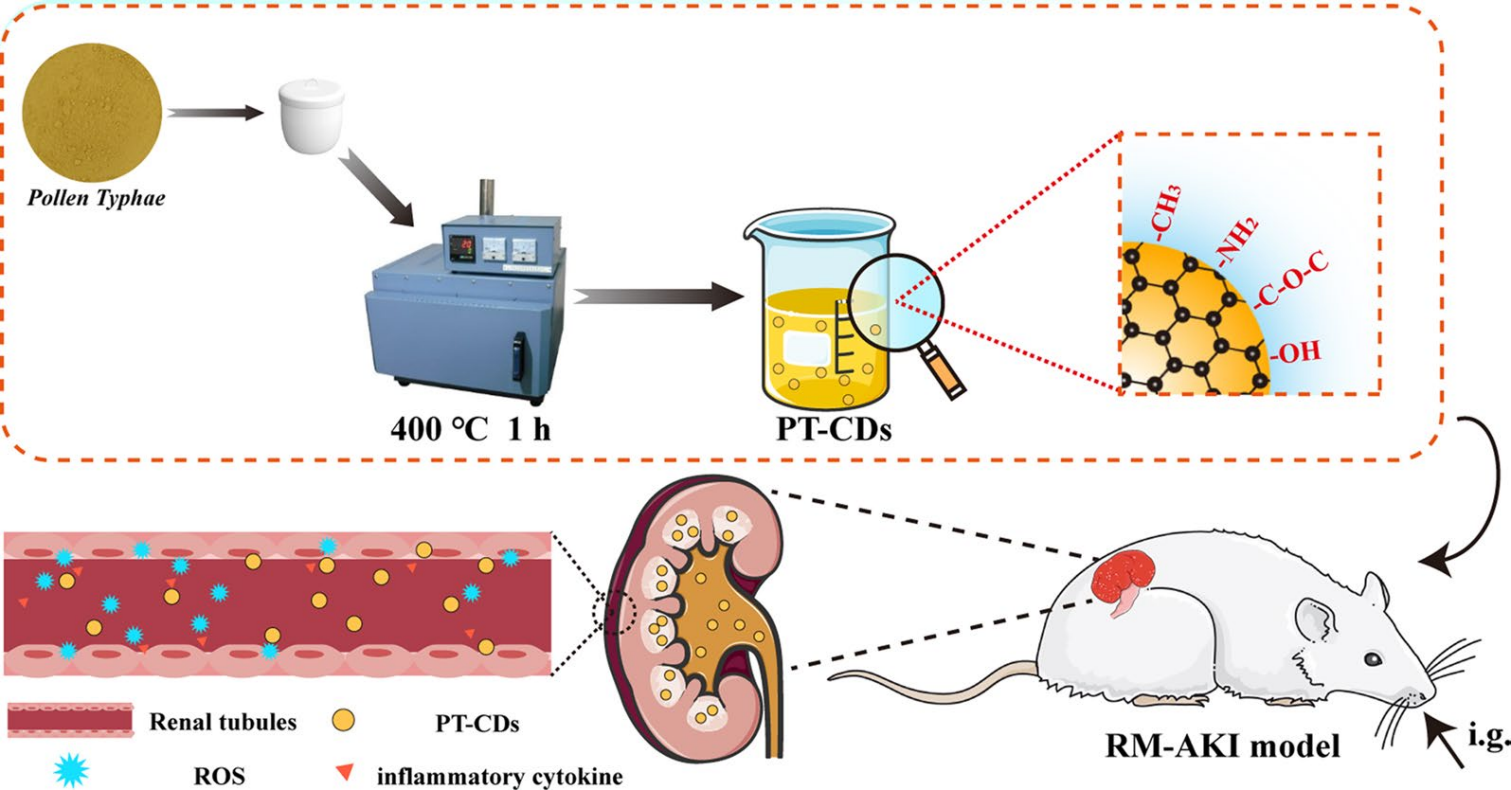

**Supplementary Information:**

The online version contains supplementary material available at 10.1186/s12951-023-01795-5.

## Introduction

Acute kidney injury (AKI) can rapidly decrease kidney glomerular filtration and accumulate metabolic waste products in the blood. It refers to a tricky clinical renal disorder for its high morbidity and mortality with an estimated 1.7 million deaths around the world each year [1–3]. The AKI induced by rhabdomyolysis, nephrotoxin (e.g., cisplatin or doxorubicin chemotherapy), ischemia/reperfusion, and sepsis primarily leads to overbalanced kidney-infiltrated reactive oxygen species (ROS) and concurrent inflammation. Aggressive increases of blood urea nitrogen (BUN) and serum creatinine (CRE) are caused by severe injuries and dysfunction of renal function units, which are considered the basic indicators of clinical diagnostics of AKI [4, 5]. Over the past few years, there is no specific treatment for this global health challenge, other than some supportive therapies (e.g., renal replacement therapies or hydration) [6]. Small molecule drugs (e.g., n-acetylcysteine, amifostine and amphotericin) have served as antioxidants to restrict AKI progression for their anti-inflammatory and antioxidative activities [4, 6]. However, low bioavailability and the potential biotoxicity attributed to the massive dose restrict the above applications to ensure the application on the clinical AKI. [7, 8] Thus, the development of a novel drug utilizing to relieve the AKI takes on a greater practical significance.

Carbon dots (CDs), a new carbon-based luminescent nanomaterial discovered in 2004, have aroused growing attention in various applications in the bioimaging, drug transport and biotherapy for their high bioactivity, photoluminescence property, low toxicity and high biocompatibility [9–12]. On the one hand, based on the ultra-small size (< 10 nm in general) and unique surface structures, many of reported CDs almost pass through the barrier systems in the state of pathological or normal, accurate in the target organ and provide specific bioactivities to the syndromes [13, 14]. The prussian blue-based CDs [15], selenium-doped CDs [16] and graphene-based CDs [17] can remove the ROS and alleviate the degree of AKI, which may be correlated with their surface structure as H-atom donors for ROS trapping [18]. On the other hand, the chemically synthesized CDs with the complex manipulation and chemical reagent contaminating properties have a certain obstruction in the clinical translation. In contrast, the biomass-based CDs have naturally aroused wide attention because of cheap carbon source, low biotoxicity, simple synthesis process, and potential application [19–21]. Moreover, the obtained CDs have a portion of the functional groups from precursors compounds preserving on their surface, thus exerting a potential effect for treating the damage [22]. Accordingly, using biomass as the novel precursors to synthesis functional CDs with high biocompatible and treatment takes on greater development and utilization significance to AKI.

Recently, the use of natural plants (herbs) with a long history in health care offers much evidence in the development of novel nanotechnology [23, 24]. In Traditional Chinese Medicine (TCM), the charcoal medicine is a series of medicines prepared from natural plants by high-temperature processing [25], while current studies of the bioactivity material basis of the above medicines are still unsatisfactory. Interestingly, considerable carbon-based nanoparticles as similar as CDs were separated from charcoal medicines, with stable carbon core, small size and considerable oxygen-rich functional groups [26], providing novel treatment ideas in the cancer-related anemia [27], psoriasis [28], ischemic stroke [29], ulcerative colitis [30], anxiety [31] and antibacterial [32, 33]. Our previous study found that CDs synthesized from *Pollen Typhae* (PT) [34] have excellent hemostatic activity through the control over coagulation parameters. Moreover, PT has a long history of clinical history. As recording in the classical TCM book “Treatise on Cold-Induced and Miscellaneous Diseases” (written in East Han dynasty, 25–220 A.D., China), the overly charred powder of PT had shown the amelioration in the kidney damage, particularly to the AKI, and were still employed in the clinical therapy. However, there is insufficient information to explain the material basis and inherent amelioration mechanism.

**Fig. 1:**
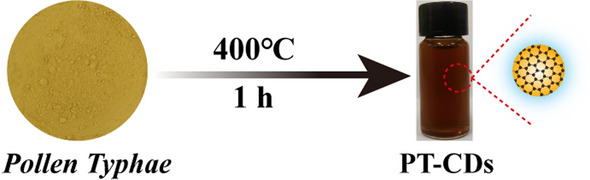
Schematic diagram of the synthesis of PT-CDs

Rhabdomyolysis (RM) was recognized as one of the main factors to induce AKI. Notably, RM-induced AKI remains a common complication of SARS-CoV-2 infection [35]. However, the available treatments by nanoparticles are most synthesized chemically [36, 37], and few relevant studies reported CDs synthesized through only natural plants to relieve RM-AKI. Herein, we firstly reported a non-toxic and functional nanoscale CD derived from PT (named PT-CDs) in specific temperature through one-step pyrolysis method dispersing without any organic solvent (Fig. 1), with controlled particle size by changing processed temperature. Besides, the above CDs have shown well regulation to kidney function indicator substances (BUN and CRE). Meanwhile, physicochemical properties of PT-CDs were characterized including morphology, optical feature, and surface structure, suggesting that the surface of PT-CDs retained partial functional groups of active pharmacophores from the precursor and carried negative charges. Moreover, intragastrical PT-CDs achieved robust amelioration to treat RM-AKI due to excellent antioxidant properties and inhibition of inflammatory factors, relieved kidney injury on day 3 of AKI rat models and promoted the recovery of the declining kidney function. This study first revelated the potential anti-AKI effect of the above ultrasmall CDs derived from natural plant, and highlights their application as a new strategy for the amelioration of AKI.

## Result

### The improved preparation conditions of PT-CDs

**Fig. 2:**
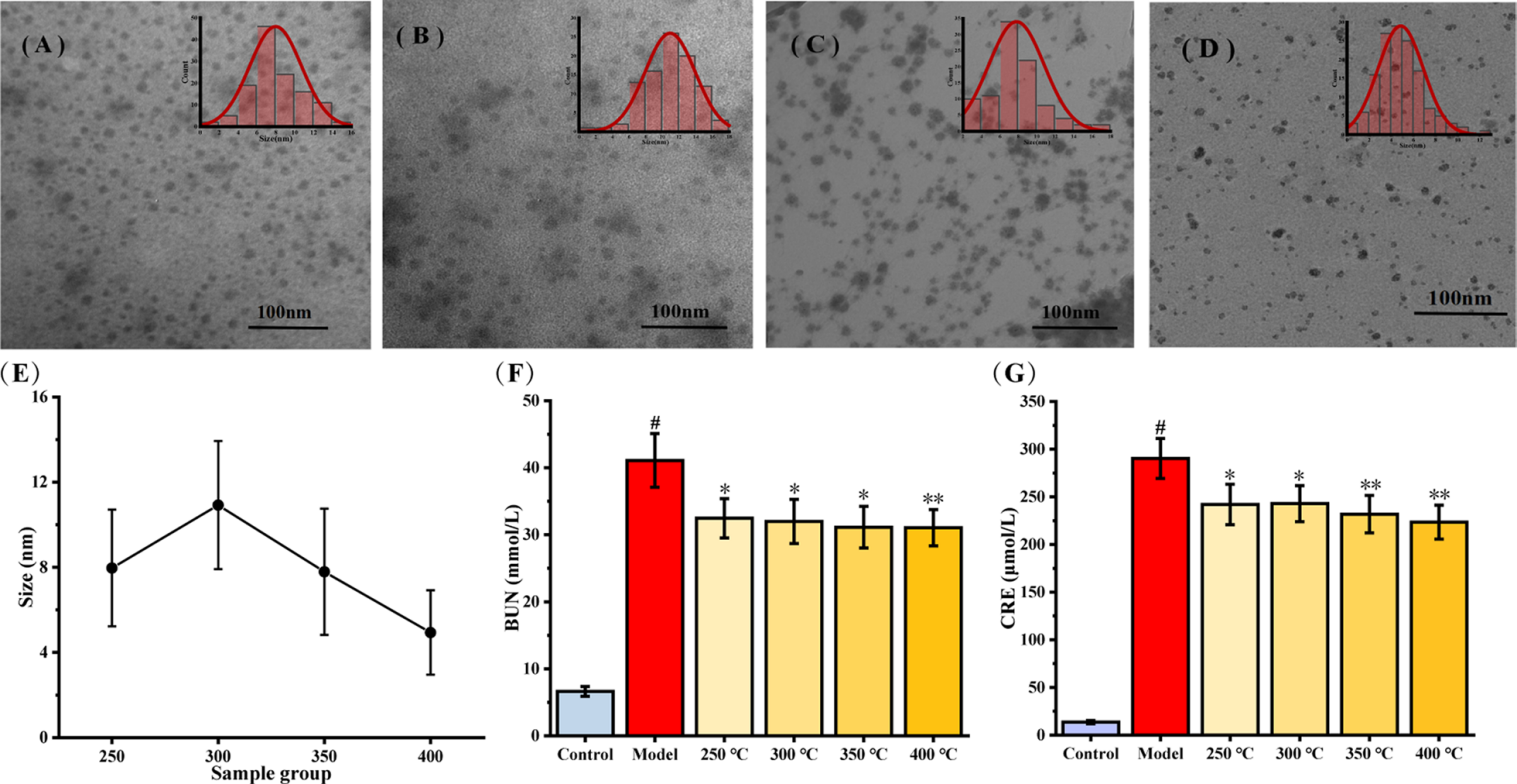
The optimize preparing conditions of PT-CDs. **A**–**D** TEM images of four kinds of PT-CDs in different temperatures (250 ℃, 300 ℃, 350 ℃ and 400 ℃, 1 h). Insets: TEM measured diameter distribution of PT-CDs. **E** Average particle size variation of PT-CDs synthesised in different temperatures. **F**, **G** Effect of PT-CDs prepared at different temperatures on the serum levels of BUN and CRE in RM-AKI model rats. **P < 0.01 and *P < 0.05 compared with model group

Ultrasmall PT-CDs were synthesized via a simple and robust preparation. The obtained CDs was identified by transmission electron microscopy (TEM) and evaluated their affect to the main kidney function. At shown in the images of TEM, the sizes of PT-CDs in 250, 300, 350 and 400 ℃ were all less than 20 nm, and the size distributions from more than 100 random CDs were ranging from 0 –14 nm, 0–18 nm, 0–19 nm and 0–11 nm, respectively (Fig. 2A–D). Besides, the average particle sizes tended to increase and then decrease, and the PT-CDs prepared at 400 ℃ had the least average particle (4.85 ± 2.06 nm) (Fig. 2E). The evaluation of kidney function and the result in Fig. 2F indicated that the PT-CDs prepared at 250 ℃ (32.46 ± 2.93 mmol/L, P < 0.05), 300 ℃ (31.98 ± 3.29 mmol/L, P < 0.05), 350 ℃ (31.13 ± 3.11 mmol/L, P < 0.05) and 400 ℃ (31.05 ± 2.70 mmol/L, P<0.01) decreased the BUN index of rats, compared with BUN of model group (41.83 ± 4.00 mmol/L). Moreover, as depicted in Fig. 2G, the PT-CDs prepared at 250 ℃ (241.95 ± 21.56 μmol/L, P < 0.05), 300 ℃ (242.85 ± 18.79 μmol/L, P < 0.05), 350 ℃ (231.75 ± 19.58 μmol/L, P < 0.01) and 400 ℃ (223.42 ± 17.90 μmol/L, P < 0.01) reduced the level of CRE compared with model group (13.58 ± 1.74 μmol/L). The concentration of BUN and CRE on the PT-CDs tended to decrease with the rise of the preparation temperature. Combining with the particle sizes and affect to the kidney function, the optimal nanoscale PT-CDs with the optimal regulation in the renal function was the procession at 400 ºC for 1 h.

### Characterization of PT-CDs

#### Morphology and DLS feature

**Fig. 3:**
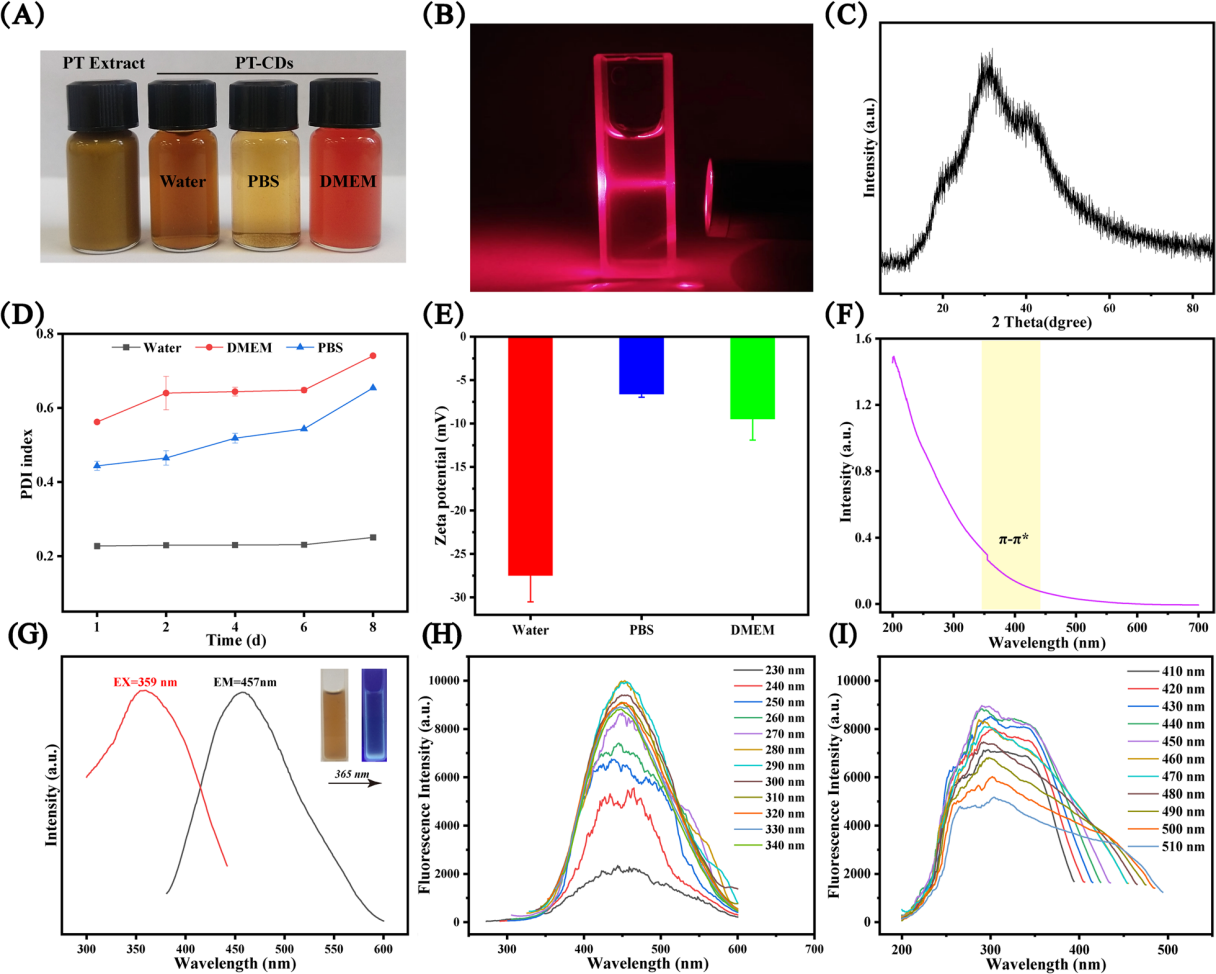
Appearance, crystalline state, optical properties and DLS assay of PT-CDs. **A** PT extracted solution and PT-CDs in different solutes (water, PBS and DMEM). **B** The Tyndall effect of PT-CDs. **C** The spera of X-ray diffractometer (XRD). **D** The colloid stability of PT-CDs dispersed in water, PBS, and DMEM measured by using dynamic light scattering (DLS). **E** The zeta-potential of PT-CDs in water, PBS and DMEM. In **D**, **E**, error bars represent the standard averaged three parallel experiments. **F** UV–vis spectrum of PT-CDs. **G** The maximum excitation and emission of PT-CDs performed by Fluorescence spectra. **H** Fluorescence spectra of PT-CDs in different excitation wavelength. **I** Fluorescence spectra of PT-CDs with different emission wavelength

HRTEM and XRD were performed to observe crystals sturcture to study the feature of PT-CDs. In the morphology image of HRTEM, PT-CDs exhibited a graphite-like crystalline structure with a lattice spacing of 0.21 nm (Additional file 1: Fig. S1), similar to the (100) diffraction facets of graphitic carbon [17]. Tyndall effect of PT-CDs solution was significant (Fig. 3B). The diffraction peak of the XRD spectrum at 32.6º confirmed the amorphous carbon structure arranged in a significantly random fashion (Fig. 3C). Next, the dispersion stability of PT-CDs were commonly examined through DLS. With the use of DLS, the colloid stability of PT-CDs in water, PBS, and DMEM at 25 ºC for 8 days was studied. The results indicated that there were no significant changes of the polymer dispersity index (PDI) in the water and increased PDI in the PBS and DMEM with 8 days (Fig. 3D). Furthermore, the surface charges were detected by measuring zeta potential. The zeta potential value was nearly − 27.5, − 6.62 and − 9.5 mV in water, PBS and DMEM, respectively, thus suggesting that the negatively charged surface of the PT-CDs (Fig. 3E).

#### Optical characterization

The UV–vis spectrum and FL spectrum have been confirmed as the main methods to characterize the optical nature of CDs. A weak absorption peak at 350 nm was identified in the UV–vis spectrum (Fig. 3F), belonging to the π-π* electric transition of PT-CDs on the skeleton [38, 39]. The max emission (EM) wavelength and excitation (EX) wavelength were at 457 nm and 359 nm, respectively (Fig. 3G), as indicated by the fluorescence emission spectra, and the QY of PT-CDs was 8.47%, which was obtained with quinine sulfate as a reference. Meanwhile, as depicted in the inset picture in Fig. 3G, the aqueous solution of PT-CDs was brown in the room light, emitting blue fluorescence under UV light (365 nm), which was due to the recombination of radiation between electrons and holes generated and trapped on the surface of the CDs [40]. With the increase of the excitation wavelength from 230 to 340 nm at 10 nm intervals (Fig. 3H), the maximum emission wavelength of PT-CDs was independent of excitation, and its fluorescence intensity tended to first increase and then decrease. Moreover, Fig. 3I also demonstrated that the maximum excitation has no significant shift from 410 to 510 nm. As revealed by the above results, the fluorescence of PT-CDs exhibits excitation dependent emission behavior, which may originate from different emission states from different surface functional groups of CDs [41].

#### Structure characterization

**Fig. 4:**
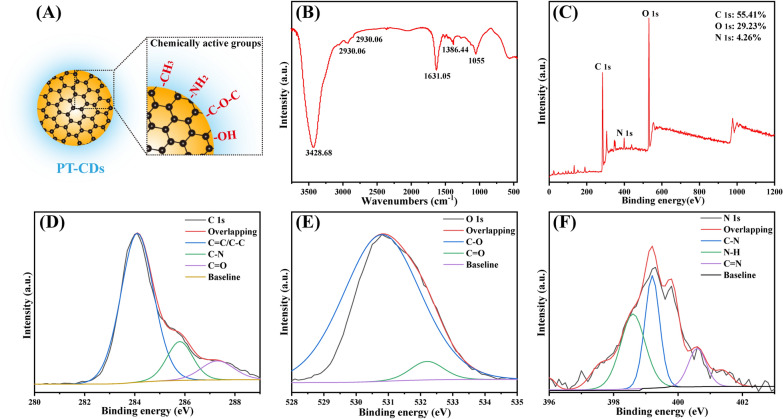
Structural characterization of PT-CDs. **A** Schematic diagram of PT-CDs with a graphite-like core and chemically active groups. **B** FT-IR spectrum of PT-CDs. **C** XPS survey spectrum of PT-CDs. **D**–**F** XPS of C 1 s, O 1 s and N 1 s, respectively

FT-IR spectra were obtained to ensure the surface structure, so as to gain more insights into the structural feature on the PT-CDs. As depicted in Fig. 4B, the FT-IR identified the strong characteristic absorption at 3428.68 cm^−1^ attributed to O–H/N–H bonding [42]. The absorption signals at 2930.06 cm^−1^ and 2852.63 cm^−1^ belonged to C–H stretching, which possibly occurred the blue shift because of the association of methyl or methylene groups with aliphatic hydrocarbons present in PT-CDs. The intense peak at 1750 cm^−1^ was related to C= O. The peak at 1377.76 cm^−1^ belonged to C–O–C stretching. And the peak located at 1055 cm^−1^ belonged to the existing of aromatic alkoxy bonds. Additionally, XPS was performed to characterize the surface elemental compositions and functional groups of PT-CDs. The XPS spectrum showed three major peaks for C (55.41%), O (29.23%) and N (4.26%) (Fig. 4C). As depicted in Fig. 4D–F, the high-resolution C1s spectrum showed significant peaks at 287.4 eV, 285.6 eV and 286.3 eV, belonging to the presence of C =O, C–N and C=C/C–C, respectively [43]. There were two peaks in O1s spectrum at nearly 532 eV and 530.8 eV, which were related to C–O and C= O groups, respectively. Furthermore, the N1s spectrum displayed peaks in 399.1 eV and 400.1 eV, belonging to the N–H bond and the C–N bond. [44] The derived functional groups (e.g., hydroxyl, amino and carbonyl [45]) were on the surface of PT-CDs, as indicated by the result of the XPS spectra, consistent with the surface groups demonstrated by FT-IR spectrum.

### Cytotoxicity detection and hemocompatibility

**Fig. 5:**
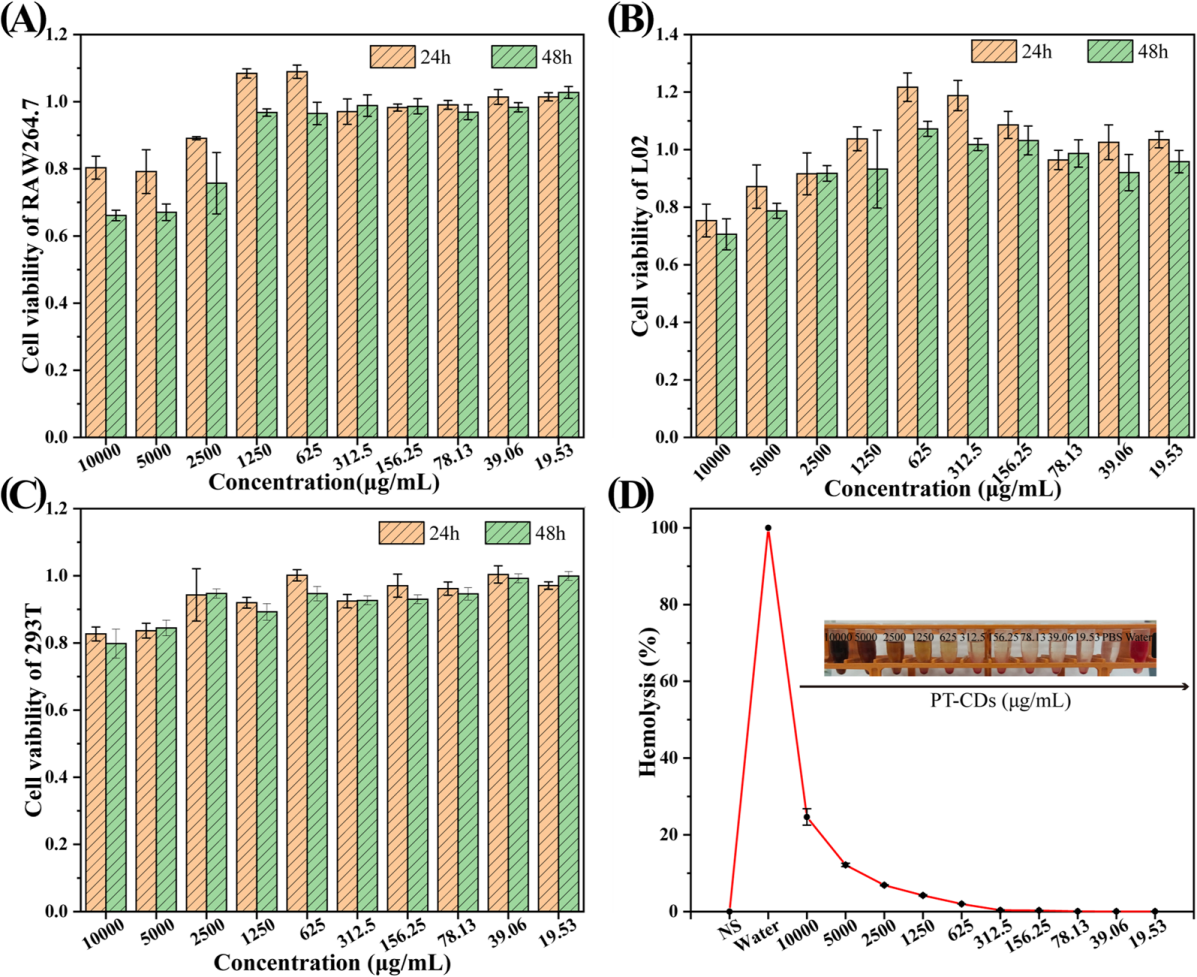
Cytocompatibility in vitro. **A**–**C** The cytotoxicity detected by CCK-8 on RAW 264.7, L02 and 293T at 24 and 48 h, respectively; **D** The Hemolytic assay of PT-CDs at different concentrations. In **A**–**D**, error bars represent the standard deviation from three repeated experiments

Due to many significant properties of potential drugs, the toxicity and biosafety of CDs are the critical part on the development of the new nanomaterials, and the cytotoxicity was determined using the CCK-8 assay. As shown in Fig. 5A–C, the concentration ranged from 19.53 to 2500 μg/mL have almost no effect on the cell viability of three cells. When the concentration of PT-CDs reached or exceed 2500 μg/mL, the survival rates of 293T cells were still up to 80% and the survival rates of RAW 264.7 and L02 cells were both up to 60%, which suggests that the low cytotoxicity of the PT-CDs. Hemolytic assay refers to the basic index to evaluate the potential hemolysis of the drug. As depicted in the Fig. 5D, PT-CDs in the respective concentration had no significant hemolytic effect to the erythrocytes. The hemolysis rates of PT-CDs were significantly lower than the internationally recognized standard (5%) from 2500 to 19.53 μg/mL, consistent with the cytotoxicity experiment. Accordingly, PT-CDs exhibited high cell compatibility.

### Biocompatibility assay

**Fig. 6:**
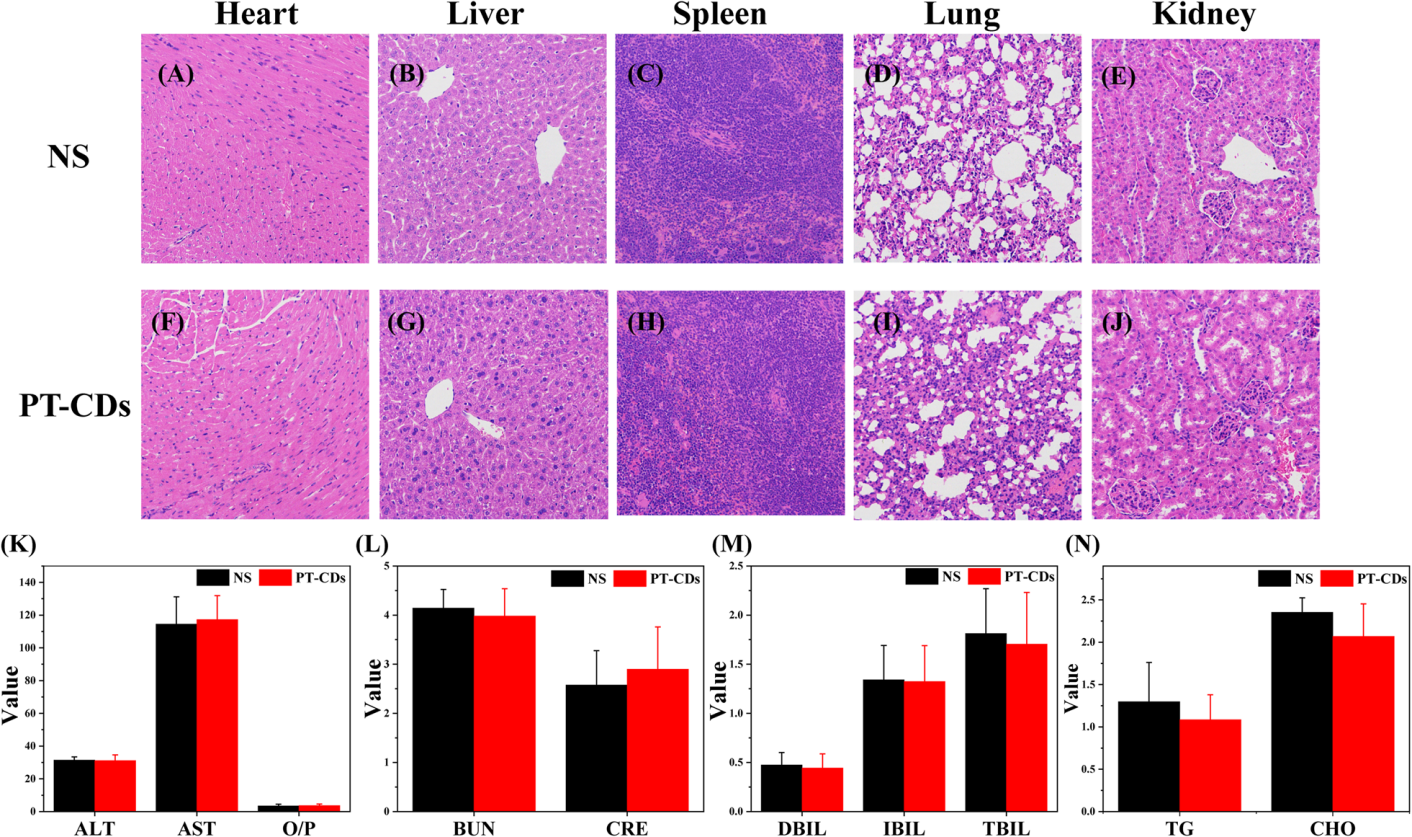
Biocompatibility in vivo. **A**–**J** H&E stained images of major organs after intragastric administration of the normal saline and PT-CDs. Scale bar: 200×. Relevant kidney and blood parameters (**K**–**N**) were measured to evaluate the toxicity of PT-CDs. In **K**–**N**, error bars represent the standard deviation from ten repeated experiments

In accordance with the biocompatibility in vivo, any potential toxicity from PT-CDs was examined by measuring relevant blood parameters and H&E staining of the major organs. KM mice were administering intragastrically with PT-CDs (30 g/kg) and then euthanized at 7-day i.p. when blood samples and organs were collected. No mice were dead in the PT-CDs infusion for the 7 days as similar as the control. As presented in Fig. 6A–J, H&E staining of the major organs identified no significant histological changes after excess PT-CDs solution ingestion in comparison with the control group. Furthermore, no toxicity from PT-CDs was identified in the kidney profile biochemical parameters in blood samples. (Fig. 6K–N) Above results all showed the low toxicity of the PT-CDs in vivo.

### The PT-CDs mitigated glycerin-induced RM-AKI model in rats

**Fig. 7:**
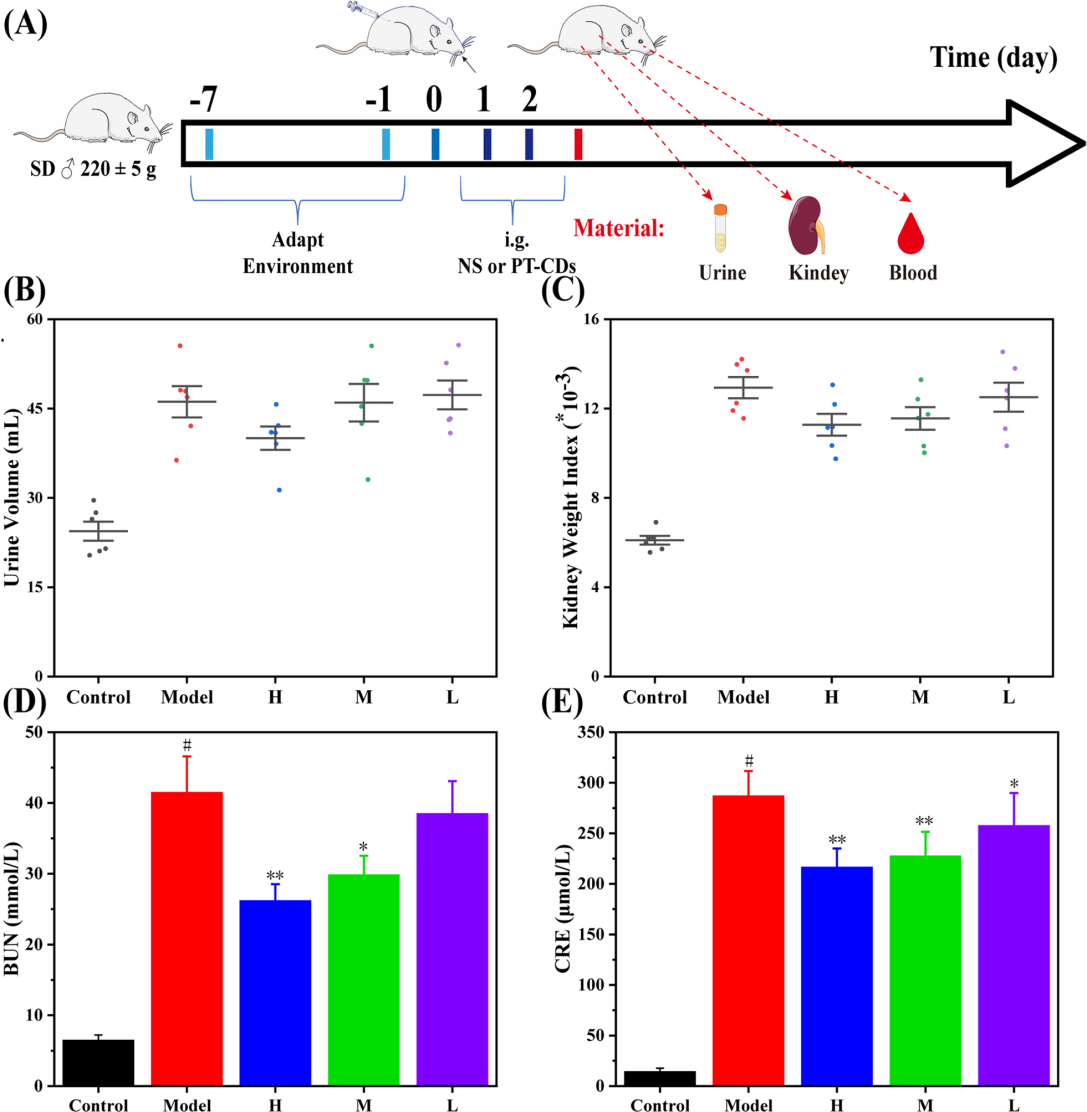
Schemes of RM-AKI model and model biomarker measurements. **A** Overview of attenuation experiments in rats. Effects of PT-CDs on **B** urine volume and **C** kidney weight index. **D**, **E** Serum BUN and CRE in rats after 2 days treatment. Each bar represnets the mean ± standard deviation (n = 10). Statistical significance: **P < 0.05, **P < 0.01 with model group

RM-AKI rat model was developed as illustrated in Fig. 7A, and the alimentation effect from PT-CDs to RM-AKI was examined by testing urine volume, kidney weight index, BUN and CRE. On the one hand, urine volume and kidney weight index both are the macro indicators to evaluate the renal function, which was shown in Fig. 7B and C. In comparison with the control group (24.41 ± 3.91 mL, 6.10 ± 0.48), urine volume and kidney weight index both had significantly elevated (46.15 ± 6.46 mL, 12.94 ± 1.16, respectively, P < 0.01), suggesting that the model was functional. Compared with those in the model group, urine volume tended to decrease from low dose group (47.29 ± 5.89 mL, P > 0.05), medium dose group (45.99 ± 7.73 mL, P > 0.05) to high dose group (40.03 ± 4.80 mL, P > 0.05). Compared with the model group, the kidney weight index of rats in the high dose group (11.28 ± 1.12) was significantly decreased (P < 0.01). Besides, both were decreased in the medium dose group (11.56 ± 1.24) amid low dose group (12.50 ± 1.59) with no statistical difference.

On the other hand, Fig. 7D and E. shows the effect of PT-CDs in the BUN and CRE on the renal function of rats with RM-AKI. The RM-AKI model has a significant increase of BUN (41.5 ± 5.09 mmol/L, P < 0.01) and CRE (287.18 ± 24.45 μmol/L, P < 0.01) compared with the levels in the control group (6.48 ± 0.74 mmol/L, 14.25 ± 3.36 μmol/L, respectively), showing good consistency with existing research [37]. Notably, high dose group and medium dose group both down-regulated the levels of BUN (26.2 ± 2.33, 29.85 ± 2.69 mmol/L, respectively) and CRE (216.5 ± 23.96, 227.58 ± 23.96 μmol/L, respectively), and the former one exerted better effect. Furthermore, BUN (38.52 ± 4.58 mmol/L, P > 0.05) and CRE (257.57 ± 32.34 μmol/L, P < 0.05) were decreased in the low dose group. Besides, PT-CDs are dose dependent in mitigating RM-AKI.

**Fig. 8:**
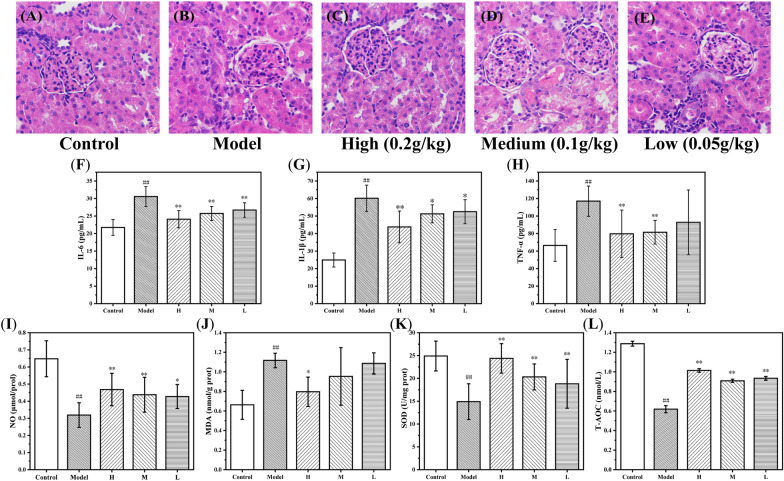
H&E staining in RM-AKI model and effects of inflammatory cytokines, myeloperoxidase activity and antioxidant levels. (A–E) Histopathological sections of kidney tissue (Scale bar: ×400): **A** control group; **B** model group; **C** high dose PT-CDs group; **D** medium dose PT-CDs group; **E** low dose PT-CDs group. Kidney concentrations of **F** IL-6, **G** IL-1β and **H** TNF-α; Effects of **I** NO, **J** MDA, **K** SOD and **L** T-AOC. Significantly different compared with the control group at ^##^P < 0.01, significantly different compared to the model group at **P < 0.01 and *P < 0.05

Hematoxylin and eosin (H&E) staining of the kidney sections were further evaluated to the morphological changes. As depicted in Fig. 8A–E, the RM-AKI model group showed considerable casts and tubular cellularity in contrast of the control group. In comparison, RM-AKI model administrated with PT-CDs had much less damage and showed less difference with the control group, especially the high dose group. The histopathological observations were identified the similar changes with the index of BUN and CRE. This evidence directly showed that PT-CDs inhibited kidney damage arising from AKI.

### Effect of PT-CDs on inflammatory cytokines

The effects of three concentrations of PT-CDs on several proinflammatory factors in the kidney tissue including IL-6, IL-1β, and TNF-α were tested in response to change of RM-AKI. Kidney IL-6, IL-1β, and TNF-α (60.13 ± 7.54, 30.55 ± 2.86 and 116.97 ± 17.29 pg/mL, respectively) levels were significantly increased in RM-AKI model in comparison with control group (P < 0.01). According to the level of IL-6, three doses of PT-CDs all showed significantly decreased relative to model group as Fig. 8F (P < 0.01). In terms of level of IL-1β as Fig. 8G, a remarkable reduction could be identified in the high dose group of PT-CDs (P < 0.01 in comparison with model group), and the other dose of PT-CDs also have a significant decline to the IL-1β (P < 0.05). In addition, both high dose group (P < 0.01) and medium dose group (P < 0.01) have a significant tendency to the level of TNF-α. Although low dose group did not show any statistical difference with the kidney TNF-α level, a significant decreasing tendency was identified in Fig. 8H. Above results all indicated the same dose dependent in different concentrations of PT-CDs to the IL-6, IL-1β, and TNF-α.

### Effect of PT-CDs on myeloperoxidase activity and antioxidant levels

Oxidative stress is one of the important processes in the occurrence and development of AKI. The redox state changes were evaluated the total antioxidant capacity (T-AOC) and antioxidant levels (NO, SOD and MDA) in the kidney tissues. (Fig. 8I–L). RM-AKI model group have a significant increase in the levels of MDA (1.12 ± 0.07 nmol/g port, P < 0.01) compared with the control group (14.91 ± 3.91 nmol/g port). Therefore, compared with the control group (NO: 0.65 ± 0.10 μmol/prot; SOD: 24.90 ± 3.27 U/mg prot; T-AOC: 0.62 ± 0.04 nmol/L), RM-AKI group caused significant decrease in the activities of NO (0.32 ± 0.07 μmol/prot, P < 0.01), SOD (14.91 ± 3.91 U/mg prot, P < 0.01) and T-AOC (0.62 ± 0.04 nmol/L, P < 0.01).

In addition, in comparison with the model group, the levels of MDA in the high (0.80 ± 0.15 nmol/g port), medium (0.95 ± 0.29 nmol/g port) and low dose (1.09 ± 0.11 nmol/g port) PT-CDs groups tended to decrease though just the high dose one achieved the statistical difference with the model group (P < 0.05). In the levels of NO, three different doses of PT-CDs groups all had the similar increase intendency similar as the dose (high and medium: P < 0.01; low: P < 0.05). Moreover, in comparison with the model group, the level of SOD was increased into the level, similar as the control group in the high dose group (24.8 ± 3.22 U/mg prot, P < 0.01), and the other dose group indicated the decrease of the SOD (medium: 20.33 ± 2.84 U/mg prot, P < 0.01; low: 18.81 ± 5.36 U/mg prot, P < 0.01). Furthermore, the levels of different concentrations PT-CDs had a significant increase in the T-AOC (high: 1.01 ± 0.02 nmol/L, P < 0.01; medium: 0.91 ± 0.02 nmol/L, P < 0.01; low: 0.94 ± 0.02 nmol/L, P < 0.01).

## Discussion

A rapid development of nanoparticle-based medicine has aroused wide attention for their unique surface structure, large surface area, and diverse physicochemical properties. Notably, the biological effects of the entry of nanomaterials into living organisms are highly complex. On the one hand, based on high surface free energy and carrying charge, nanoparticles easily bind to proteins to form protein crowns [46], especially in oral exposure, which may fundamentally alter the nanoparticles in the biological capacity in the organisms [47]. On the other hand, nanomaterials are related with the complex physicochemical properties, which affects receptor identification or biological reactions in different degree. The above properties constitute the basic active variation of nanomaterials in vivo. As the novel carbon-based nanomaterials, the unique inherent properties of CDs have driven the attention of scientists, however, related study is still in its infancy, with plentiful underlying bioactivities and application prospects. Against this status quo, CDs with novel bioactivity were merited to be designed and developed to advance broaden their application in bioactive fields.

Natural plant-derived CDs, a major precursor of green biomass with rich chemical composition, have aroused wide attention for green sources and remarkable bioactivities [21, 48]. Nowadays, bottom-up methods have aroused the interest of scientists for their double advantage of being straightforward and economical [27, 49], such as hydrothermal method and pyrolysis method. Several current plant-derived CDs were prepared with the pyrolysis method (Additional file 1: Table S1), and showed remarkable bioactivities for treating diseases. In comparison with the common hydrothermal method, this method still has higher temperatures but shorter time, which can meet the requirements of large-scale production [50], while the obtained CDs exhibit uniform particles distribution and smaller particle size [19], with certain fluorescence and lower QY(≤ 10%). Furthermore, our team previously illuminated the treatment of CDs synthesized using the high-temperature method in hemostatic and ulcerative diseases, with no significant undesirable affect in cells. The above information provides related evidence to get PT-CDs from charred PT to investigate its efficacy.

As classical herbs recording in the 2020 Pharmacopoeia of the People’s Republic of China (PPRC), PT and its charred product PTC both have widely clinic utilization. Based on the larger surface area, PT have easily transformed into CDs in high temperature compared with other biomass precursors. There are lacking quantitative description for the charred medicine in the clinical reports due to the technical limitation, and existing information was only recorded in terms of morphology (e.g., color). Our previous study has suggested that the CDs from same precursors observe different bioactivities at the different temperatures, such as that *Atractylodes macrocephala*-based CDs [51] prepared at different temperatures have anti-gastric ulcer activity in different degree, and *Phellodendri Chinensis Cortex*-based CDs [28, 52, 53] prepared in 350 ℃ and 400 ℃ showed hemostasis and anti-psoriasis effect, respectively. Next, the results of a series of experiments indicated that with an increase at the treatment temperature from 250 to 400 ℃, the color of the charred herbal appearance darkened as follows (Additional file 1: Fig. S2), the carbonization degree of the PT tended to be increased, whereas the size distribution of obtained CDs changed, and the activity of anti-AKI effect tended to increase, with the optimal anti-AKI efficacy and the least size at 400 ℃.

Moreover, many of characterization were adopted to explore the surface structure and feature to PT-CDs in optimal conditions. The HPLC was used and observed no significant peak in all retention time of PT-CDs solution (Additional file 1: Fig. S3), performing a similar result as the previous study [34], which can exclude the effect from the entrained small molecular compounds like isorhamnetin-3-o-neohesperidoside or typhaneoside [54]. The changes of PT-CDs on the size and morphology by TEM are similar to the reported CDs [27, 55]. As the previously reported [22, 56], the ultra-small size (4.85 ± 2.06 nm) in the optimal sample may pass through the glomerular filtration barrier (GFB) and further accumulate, which suggests the possible mechanism in the kidney function by the PT-CDs. Furthermore, derived functional groups on the surface of PT-CDs were demonstrated by FT-IR and XPS spectra, and the above active groups including –OH, –C =O and –C–O–C can be identified. The above multiphoton activation of the above oxygen-containing functional groups can contribute to the photoluminescence of CDs. [57]. Besides, a wide variety of functional groups may provide explanation for the prominent amelioration against RM-AKI bioactivity by PT-CDs.

The biosafety of nanoparticles has been confirmed as one of the obstacles to clinical translation, with side effects and inappropriate organ accumulation potentially affecting normal physiological functions [58], which should incorporate more biosafety considerations to evaluate. The PT-CDs in this study have low cytotoxicity, weak hemolytic and high biocompatibility due to the green synthesis process, carried negative charges and abundant surface groups, thus providing basic biosafe information for future application. Meanwhile, the stable PDI in different matrices also supports that this CDs cannot undergo malignant aggregation and have a continuous stability. The above results suggest the suitability of PT-CDs in the biomedical application.

In addition, the etiology of AKI is still complicated and not full elucidated till now, existing evidence proposed that the regulation of disfunctional renal function is one of the key way in AKI mitigation [4]. In this study, RM-AKI model had significant increases of BUN and CRE in serum levels, which indicates the functional recapitulation of the acute kidney injury model of rats, consistent with the literature. [37] Furthermore, all doses of PT-CDs reduced the BUN and CRE, particularly the high dose, indicating that PT-CDs can regulate the levels of BUN and CRE and have alleviate effect in RM-AKI. Moreover, several experiments were performed to evaluate the change of the kidney function. On the macro level, in accordance with RM-AKI, changes in urine volume and differences in kidney indexes were more common. Compared with the model group, urine volume and kidney indexes tended to be decreased through PT-CDs administration, suggesting the effect of PT-CDs in facilitating the kidney function recovery. At the micro level, exfoliated renal tubular epithelial cells, considerable casts, and inflammatory cell infiltration were indicated in the pathological sections of rat kidney tissue. Compared with the model groups, different doses of PT-CDs had the alleviation effect, as indicated by the results of the experiment. The above results have proven that PT-CDs can mitigate the damage of RM-AKI and recovery kidney function.

The significant changes in inflammatory reaction have been identified in the development of RM-AKI. AKI-induced activation of inflammatory cells in kidney leads to the overexpression and release of large amounts of cytokines, including IL-6, IL-1β, and TNF-α, resulting in the aggravated damage [59]. As revealed by several existing studies, the up-regulated levels of IL-6 will affect the ability of erythrocytes to carry oxygen, thus causing hypoxemia, or even rhabdomyolysis [60]. PT-CDs administrations are capable of down-regulating the levels of the IL-6, IL-1β, and TNF-α to inhibit inflammatory reaction. The possible reason for this finding is that the intervention of PT-CDs inhibited the increase of neutrophils in renal tissue while reducing their tissue chemotaxis and restrained kidney tissue harm.

Oxidative stress takes on a critical significance to the development of AKI. Since myoglobin produced by rhabdomyolysis enters the kidney, considerable ROS is metabolized to produce lipid peroxidation, thus increasing oxidative damage and inducing inflammatory responses [61, 62]. MDA and SOD are important indicators of the level of oxidative stress, representing the degree of lipidation of oxygen radicals and the activity of antioxidant enzymes, respectively [63, 64]. The improper expression of NO also increases oxidative stress [65]. PT-CDs intervention is capable of significantly increasing total antioxidant capacity, up-regulating the level of NO and SOD, and down-regulating the level of MDA, as revealed by the results of this experiment. The possible reason for this is the free radical scavenging ability of the oxygen-containing groups (e.g., –OH on CDs surface), suggesting that PT-CDs are capable of reducing the ROS, inhibiting free radical lipid peroxidation, and mitigating kidney damage. There are no significant gap in antioxidant ability between PT-CDs with other reported anti-AKI nanoparticles [16, 37, 66], suggesting the PT-CDs derived from natural plants have potential to be a substitute to the above materials.

The mechanism by which PT-CDs decrease AKI effect may prevent kidney lipid peroxidation, thus mitigating damage to kidney cells, whereas the underlying antioxidant mechanism of PT-CDs requires in-depth studies.

## Methods

### Chemicals

*Pollen Typhae* (PT) originated from Beijing Qiancao Herbal Pieces Co. Ltd. (Beijing, China) and had passed quality verification by the National Medical Products Administration (China). Subsequently, all purchased herbal materials were authenticated by Dr. Yan Zhao from the School of Traiditional Chinses Medicine, Beijing University of Chinese Medicine. Dialysis bags were provided by Beijing Ruida Henghui Technology Development Co., Ltd. (Beijing, China). The kits of IL-6, IL-1β, TNF-α, nitric-oxide (NO), superoxide dismutase (SOD), malondialdehyde (MDA) and total antioxidant activity (T-AOC) all were purchased from Cloud-Clone Corp. (Wuhan, China). All other analytical grade chemicals and reagents were offered by Sino-pharm Chemical Reagents Beijing (Beijing, China). All experiments were performed with deionized water (DW).

### Animals

Male SD rats (weighing 220 ± 5 g) and male Kunming mice (weighing 25.0 ± 2.0 g) were purchased from the Laboratory Animal Center of Si-Beifu (Beijing, China), with acclimatizing under 12 h/12 h light and dark cycle at a controlled temperature of 24.0 ± 1.0 ℃, and provided with standard chow and water ad libitum, and all animals were adeptly fed for 7 days. All animal management and experiments were approved by the Committee of Ethics of Animals Experimentation of the Beijing University of Traditional Chinese Medicine.

### Synthesis of PT-CDs

PT-CDs were synthesized using a simple one-step pyrolysis method [51]. In brief, 180 g PT powder was placed in a sealed crucible, and the crucible with PT powder was calcined at different temperatures (250 ℃, 300 ℃, 350 ℃ and 400 ℃) for 1 h in a muffle furnace (TL0612 muffle furnace; Beijing ZhongKe Aobo Technology Co., Ltd; Beijing China). Next, the 100 g of carbonized PT powder was boiled three times with 1000 mL DW for 1 h each time. Furthermore, with filtering through 0.22 μm microfiltration membrane, the concentrated dark brown filtrate was dialyzed with DW for 72 h (MWCO = 1000 Da), the DW was replaced every 8 h in the above period and centrifuged at 11,000 rpm/min for 30 min to separate the insoluble residue. The obtained PT-CDs solution was stored at 4 ℃.

### Characterization of PT-CDs

The fingerprint analysis of PT-CDs was conducted using HPLC (Agilent LC-1260, Agilent Technologies Inc., Waldbronn, Germangy) that was equipped with the Reliasil-C18 column (250 mm × 4.6 mm × 5 μm, Orochem, IL, USA). In accordance with the previous report, the modified gradient elution process with 0.1% methane acid (phase A) and acetonitrile (phase B) at a constant flow rate of 1.0 mL/min was elucidated as follows: 0–40 min, 87–80% A; 40–50 min, 80–75% A; 50–70 min, 75–50% A. The column temperature was 25 ℃, the injected sample quantity was kept at 10 mL, the detection wavelength was set as 248 nm and all sample were filtered with a 0.22 μm cellulose membrane (Jin Teng, Tianjin, China) before application. The morphology, size, and atomic lattice fringes of PT-CDs were obtained through transmission electron microscopy (TEM; Tecnai G220, FEI Company, Hillsboro, OR, USA) and high-resolution TEM (HRTEM; JEN-1230, Japan Electron Optics Laboratory, Tokyo, Japan). The fluorescence phenomenon of PT-CDs was recorded under natural light and 300 nm UV light. The PL excitation and emission spectra of the prepared PT-CDs were examined using a fluorescence spectrophotometer (F-4500, Hitachi, Tokyo, Japan). The UV–vis absorption spectra were obtained using UV–vis absorption spectrophotometer (CECIL, Cambridge, UK). The structure details of PT-CDs were studied by using the Fourier transform-infrared (FT-IR) spectrometer (Thermo Fisher, CA, USA) at the range of 400–4000 cm^−1^ and X-ray photoelectron spectroscopy (XPS, ESCALAB 250 Xi; Thermo Fisher Scientific, Waltham, MA, USA) with a monochromatic Al Kα X-ray source. The PT-CDs were dispersed in DW and examined using a Zetasizer Nano ZSE (UK) performing dynamic light scattering at 25 ºC. Furthermore, the zeta potential and polydispersity index (PDI) of PT-CDs were examined using dynamic light scattering (DLS).

### Quantum yield of PT-CDs

Quinine sulphate [quantum yield (QY), 54% in 0.1 M sulfuric acid (H_2_SO_4_)] served as a reference to obtain the QY of the PT-CDs, which is written as Eq. (1):

1 $${Q}_{CDs}={Q}_{R}\times \frac{{I}_{CDs}}{{I}_{R}}\times \frac{{A}_{R}}{{A}_{CDs}}\times \frac{{\eta }_{CDs}^{2}}{{\eta }_{R}^{2}}$$

where Q denotes QY; I represents the examined comprehensive emission intensity; A expresses the absorbance at the excitation wavelength; η is the refractive index of the solvent. CDs and R represent the PT-CDs and reference, respectively. A_R_ and A_CDs_ were kept to be lower than 0.05 to reduce the effect of reabsorption.

### Biosafety evaluation

#### Cytotoxicity assays

CCK-8 has been widely adopted to evaluate the cytotoxicity in the cytotoxicity assays of nanomaterials. The RAW 264.7 mouse macrophage, Human L-02 hepatocyte, and embryonic kidney 293T cell line were placed at 2 × 10^4^ cells per well in a 96-well plate and then incubated for 24 h at 37 ℃ in 5% CO_2_ atmosphere, respectively. Subsequently, both cell types were administrated with 100 μL of different concentrations (10,000, 5000, 2500, 1250, 625, 312.5, 156.25, 78.13, 39.06, and 19.53 μg/mL) of the PT-CDs solution in serum-free medium and then incubated for 24 h or 48 h. Subsequently, 10 μL CCK-8 was added, and then the samples were incubated for another 4 h. Furthermore, the control cells were administrated with above media. The absorbance of the respective well was recorded at 450 nm using a microplate reader (Biotek, USA). Cell viability (%) is written as Eq. (2):

2 $$\mathrm{Cell \ viability}\left(\mathrm{\%}\right)= \frac{{\mathrm{A}}_{\mathrm{a}}-{\mathrm{A}}_{\mathrm{c}}}{{\mathrm{A}}_{\mathrm{b}}-{\mathrm{A}}_{\mathrm{c}}}\times 100$$

Aa, Ab, and Ac represent the absorbance of the experimental, control, and the blank groups, respectively.

#### Hemocompatibility in vitro

The hemocompatibility of the nanoparticles was examined through hemolysis test. First, 1 mL of blood of plasminogen removal rats was diluted with 0.9% NaCl solution and then centrifuged at 9000 rpm at 4 ℃ for 15 min till erythrocytes were obtained. 10% suspension of the obtained erythrocytes was prepared and then mixed with 0.9% NaCl solution (blank control group), deionized water (positive group), and PT-CDs sample at different concentrations (19.53, 39.06, 78.13, 156.25, 312.5, 625, 1250, 2500, 5000, and 10,000 μg/mL). After the incubation for 4 h in the incubator at 37 ℃, the supernatant was separated at 3500 rpm for 10 min, and the absorbance was monitored at 570 nm using the microplate reader. Hemolysis rate (%) is expressed as Eq. (3):

3 $$\mathrm{Hemolysis\ rate}\left(\mathrm{\%}\right)= \frac{{\mathrm{A}}_{\mathrm{S}}-{\mathrm{A}}_{\mathrm{b}}}{{\mathrm{A}}_{\mathrm{P}}-{\mathrm{A}}_{\mathrm{b}}}\times 100$$

where, A_s_, A_b_ and A_P_ denote the absorbance values of sample, blank control and positive control, respectively.

#### Biosafety evaluation in vivo

The plasma was collected from orbital sinus of KM mice (n = 10) after i.g. 0.6 mL PT-CDs of 30 g/kg and normal saline solution for 7 days (i.g. twice per day). Moreover, the main organs were acquired (e.g., liver, spleen, kidney, heart, and lung). Cereal urea nitrogen (BUN), serum creatinine (CRE), third transaminase (ALT), aspartate aminotransferase (AST), P/O ratio (P/O), total bilirubin (TBIL), direct bilirubin (DBIL), indirect bilirubin (IBIL), triglycerides (TG) and total cholesterol (TC) level were examined using an automatic biochemical analyzer (AU480, USA) based on the result of the analysis of the collected plasma. Furthermore, the main organs cut into sections were stained and identified using an optical microscope.

### *AKI treatment studies *in vivo

#### The renal function effect of PT-CDs prepared at different temperatures

After the mainly renal function effect was evaluated as the elevation index, the improved temperature condition of PT-CDs were examined. Animals were randomly assigned to six groups, including control group (NS, 2.2 mL, i.g.), model groups (NS, 2.2 mL, i.g.), and PT-CDs as-prepared at different temperatures (250, 300, 350 and 400 ℃, 1 g/kg, i.g.). 10 mL/kg of 50% glycerol was intramuscularly given to both hind limbs of rats equally of the model group and the PT-CDs groups produced at different temperatures (250, 300, 350 and 400 ℃). Vacuum blood collection tubes (Becton Dickinson Medical Instrument Co. Ltd, Shanghai, China) and blood taking needles (Shangdong Junnuo co., Ltd, Heze, China) were adopted to collect blood samples through the abdominal aorta. After maintaining at 4 ℃ for 3 h, the serum samples were separated from the whole blood at 3000 rpm for 30 min, and an automatic biochemical analyzer was employed to examine BUN and CRE concentrations.

#### The treatment experiment of RM-AKI model by PT-CDs

After depriving of water but giving food ad libitum for 24 h, the male SD rats were randomly divided into five groups (n = 6): (1) control group; (2) rhabdomyolysis-induced AKI model (RM-AKI) group; (3) RM-AKI model + PT-CDs high dose (H) group; (4) RM-AKI model + PT-CDs medium dose (M) group; (5) RM-AKI model + PT-CDs low dose (L) group. Groups (2)–(5) were mounding as the mentioned above. The rats were then given access to water and food, and administering intragastrically with normal saline, PT-CDs (H) (0.2 g/kg), PT-CDs (M) (0.1 g/kg) and PT-CDs (L) (0.05 g/kg) per 12 h. At the end of the experiment after 48 h, rats were anesthetized with pentobarbital and sacrificed to collect blood and kidneys. The serum was isolated as the above methods and the related kidney biochemical indicators (BUN and CRE) were determined as the above methods. All animal studies and procedures were approved by the Institutional Animal Care and Use Committee of Beijing University of Chinese Medicine and were carried out in accordance with all guidelines and regulations.

#### Haemotoxylin and eosin (H&E) staining of kidney sections

After observing and taking pictures of kidney tissue, part of the kidney was fixed in 4% paraformaldehyde solution, dehydrated in gradient ethanol, embedded in paraffin, sliced into 5 μm thick sections, and stained with Haematoxylum and eosin (H&E). The stained sections were photographed and identified under a microscope at magni-fictions magnifications of 200 × .

#### Measurement of myeloperoxidase activity and antioxidant levels

The right kidneys originating from different groups preserved in the − 80 ℃ were homogenized with PBS on ice and then centrifuged at 10,000 rpm for 15 min. The supernatants were collected to calculate the content levels of NO, SOD, MDA and T-AOC using the respective kits in accordance with the manufacture’s instruction.

#### Detection of cytokines in Kidney

The remaining kidney tissues were collected and then stored at – 80 ℃ till the application in the following procedures. After being homogenized with PBS on ice and centrifuging at 10,000 rpm, the levels of IL-6, IL-1β, and TNF-α in kidney from rats were examined through ELISA (Cloud-Clone Technology co., Ltd., Wuhan, China) in accordance with the manufacturer’s instructions.

### Statistical analysis

The statistical analysis was conducted using the SPSS 25.0 statistical analysis software (SPSS, version 16.1, Chicago, IL, USA). The one-way analysis of variance (ANOVA) was employed for the multiple comparisons (P < 0.05) followed by the least significant difference test, and the data were expressed as the mean standard deviation (SD).

## Conclusion

In brief, novel CDs with ultra-small size and high biocompatible were successfully synthesized and segregated based on one-step pyrolysis of *Pollen Typhae*. Compared with the regulation ability of kidney functions of the above CDs synthesized at different temperatures, PT-CDs prepared in 400 ºC were selected as the optimal one with least size and best regulation of renal function for further study. Active functional groups from natural plant precursors and negative charges endow their distinctive biologic activities with no additional bioactive group modifications. This study is the first to demonstrate that PT-CDs alleviated remarkably rhabdomyolysis-induced AKI under no systemic toxicity, and the underlying basic mechanism of that effect is associated with mitigating the kidney functional damage, decreasing the pro-inflammatory factors, and improving antioxidant ability. This study provides new insights into the carbonization process of pure plant in the preparation of carbon dots, and we believe that the green, inexpensive and safer PT-CDs will provide excellent promise for the development of novel AKI drugs.

## Supplementary Information

**Additional file 1: Table S1.** Compared with plant-derived CDs for optical properties and applications. **Figure S1.** The HRTEM image of PT-CDs in 400 ℃. **Figure S2.** The appearance of PTC in different temperature (250 ℃, 300 ℃, 350 ℃ and 400 ℃). **Figure S3.** The image of HPLC assay on PT-CDs.

## Data Availability

Data sharing is applicable to this article.
