# Supplementary material for: Ultrasmall and highly biocompatible carbon dots derived from natural plant with amelioration against acute kidney injury
Source: J Nanobiotechnology. 2023 Feb 23;21:63. doi: 10.1186/s12951-023-01795-5 (PMC9946873; doi:10.1186/s12951-023-01795-5)
Supplement: Supplementary file 1 — Additional file 1: Table S1. Compared with plant-derived CDs for optical properties and applications. Figure S1. The HRTEM image of PT-CDs in 400 ℃. Figure S2. The appearance of PTC in different temperature (250 ℃, 300 ℃, 350 ℃ and 400 ℃). Figure S3. The image of HPLC assay on PT-CDs. [file 12951_2023_1795_MOESM1_ESM.docx]

Additional file

**Ultrasmall and Highly Biocompatible Carbon Dots Derived from Natural Plant with** **Amelioration Against Acute Kidney Injury**

Xiaoke Wang^1#^, Tong Wu^2#^, Yingxin Yang^3^, Long Zhou^3^, Shuxian Wang^3^, Jiaxing Liu^4^, Yafang Zhao^3^, Meiling Zhang^5^, Yan Zhao^3^, Huihua Qu^3,6*^, Hui Kong^3*^, Yue Zhang^7*^

*^1^ Encephalopathy Hospital, The First Affiliated Hospital of Henan University of Chinese Medicine, Zhengzhou 450000, China.*

*^2^ School of Chinese Materia Medica, Beijing University of Chinese Medicine, Beijing 100029, China.*

*^3^ School of Traditional Chinese Medicine, Beijing University of Chinese Medicine, Beijing 100029, China.*

*^4^ Third Affiliated Hospital, Beijing University of Chinese Medicine, Beijing 100029, China.*

*^5^ Key Laboratory of Chinese Internal Medicine of the Ministry of Education, Dongzhimen Hospital Affiliated to Beijing University of Chinese Medicine, Beijing 100020, China.*

*^6^ Center of Scientific Experiment, Beijing University of Chinese Medicine, Beijing 100029, China.*

*^7^ School of Life Science, Beijing University of Chinese Medicine, Beijing 100029, China.*

# These authors contributed equally.

*Corresponding authors. Tel +86 10 64286705; Fax +86 16 4286821

1. mail addresses:

quhuihuadr@163.com (Huihua Qu); doris7629@126.com (Hui Kong); 201801024@bucm.edu.cn (Yue Zhang);

**Table S1.** Compared with plant-derived CDs for optical properties and applications.

| **No.** | **Name of Plant precursors** | **Synthetic methods** | **Optical properties** | | | **Application** | **Ref.** |
| --- | --- | --- | --- | --- | --- | --- | --- |
|  |  |  | **EM/EX*** | **QY** | **Luminescence** |  |  |
| 1 | *Artemisiae argyi folium* | One-step pyrolysis  (350 ºC for 1 h) | EM: 410 nm  EX: 322 nm | 0.19% | Blue light | Anti-frostbite effect | [1] |
| 2 | *Paeonia radix alba* | One-step pyrolysis  (350 ºC for 1 h) | EM: 411 nm  EX: 320 nm | 5.34% | No description | Hepatoprotective Effect | [2] |
| 3 | *Atractylodes macrocephalae rhizoma* | One-step pyrolysis  (350 ºC for 1 h) | No done | No done | Blue-green light | Anti-gastric ulcers effect | [3] |
| 4 | *Juncus medulla* | One-step pyrolysis  (350 ºC for 1 h) | EM: 447 nm  EX: 336 nm | No done | No description | Hemostasis/Hepatoprotec-tion | [4] |
| 5 | *Zingiberis rhizoma* | One-step pyrolysis  (350 ºC for 1 h) | EM: 470 nm EX: 357 nm | 5.20% | No description | Analgesia | [5] |
| 6 | *Phellodendri chinensis cortex* | One-step pyrolysis  (400 ºC for 1 h) | EM: 445 nm EX: 330 nm | 5.63% | No description | Treatment of psoriasis /immunoregulation | [6] |
| 7 | *Phellodendri chinensis cortex* | One-step pyrolysis  (350 ºC for 1 h) | No done | No done | No description | Treatment of Dein-*agkistrodon acutus* venom-induced acute kidney injury | [7] |
| 8 | *Phellodendri chinensis cortex* | One-step pyrolysis  (350 ºC for 1 h) | EM: 438 nm EX: 363 nm | 9.62% | Blue light | Hemostasis | [8] |
| 9 | *Lonicerae japonicae flos* | One-step pyrolysis  (350 ºC for 1 h) | EM: 464 nm EX: 384 nm | 0.50% | No description | Anti-inflammatory effect/ alleviating fever | [9] |
| 10 | *Schizonepetae spica* | One-step pyrolysis  (350 ºC for 1 h) | EM: 454 nm EX: 355 nm | 2.26% | No description | Hemostasis | [10] |
| 11 | *Typhae pollen* | One-step pyrolysis  (350 ºC for 1 h) | EM: 442 nm  EX: 352 nm | No done | Blue light | Hemostasis | [11] |
| 12 | *Aurantii fructus immaturus* | One-step pyrolysis  (350 ºC for 1 h) | EM: 469 nm  EX: 380 nm | 7.20% | Blue light | Anti-gouty effect | [12] |
| 13 | *Armeniacae Semen Amarum* | One-step pyrolysis  (300 ºC for 1 h) | EM: 436 nm EX: 353 nm | 3.17% | No description | Treatment of acute lung injury | [13] |
| 14 | *Jujubae frucrus* | Hydrothermal  (200 ºC for 6 h) | EM: 440 nm  EX: 360 nm | No done | Blue light | Treatment of cancer-related anemia | [14] |
| 15 | *Persicae semen /Carthami flos* | Hydrothermal  (240 ºC for 16 h) | EM: 434.6 nm EX: 361.6 nm | 3.84% | Blue light | Traumatic brain injury | [15] |
| 16 | *Carica papaya leaves* | Sand bath  (180 ºC for 24 h) | EX: 440 nm  EM: 530 nm | No done | Blue light | Antioxidant and anti-inflammatory activities | [16] |
| 17 | *Typhae pollen* | One-step pyrolysis  (400 ºC for 1 h) | EM: 457 nm  EX: 359 nm | 8.47% | Blue light | Treatment of rhabdomyolysis-induced acute kidney injury | This work |

**
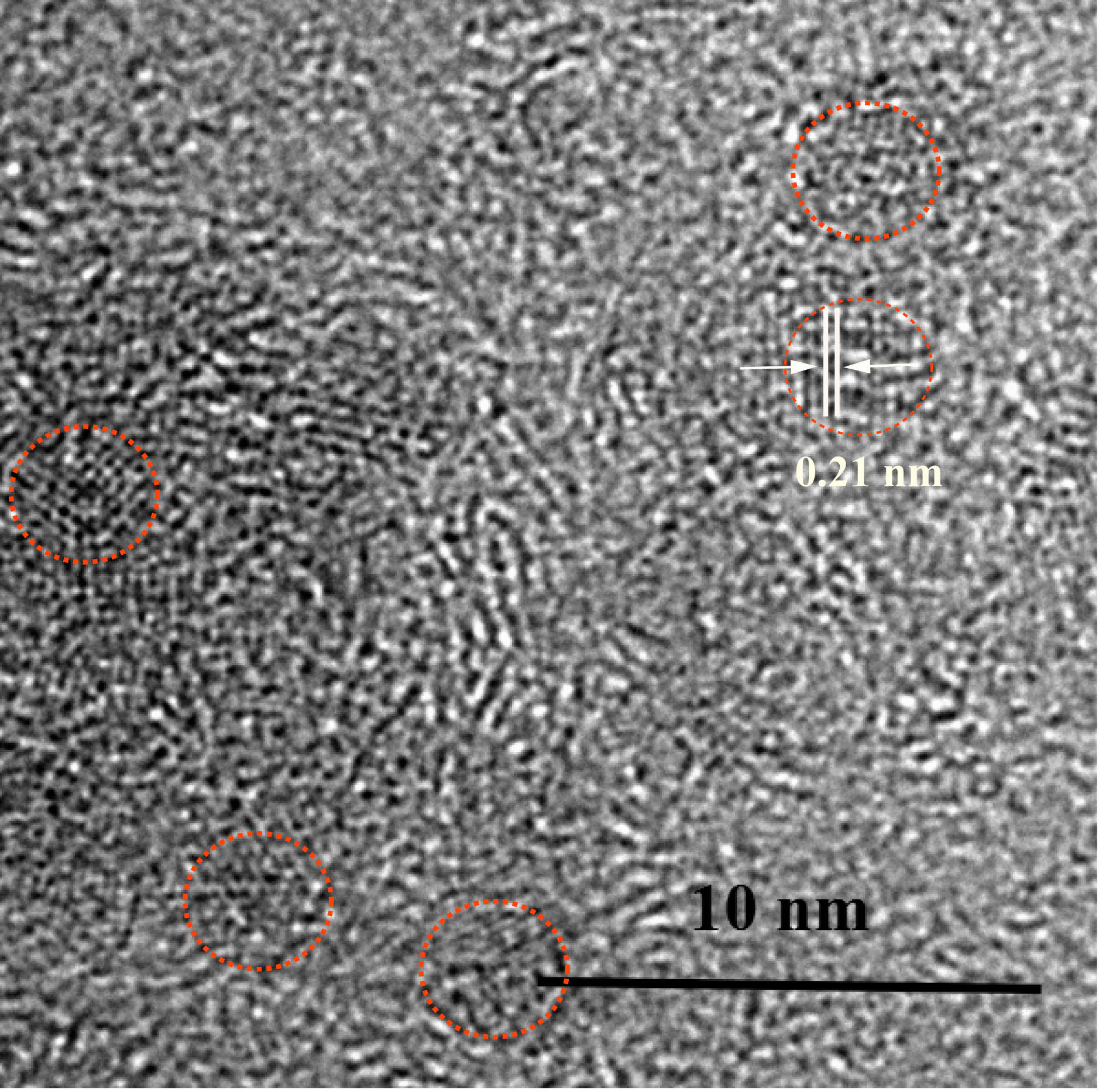
**

**Fig. S1** The HRTEM image of PT-CDs in 400 ℃.


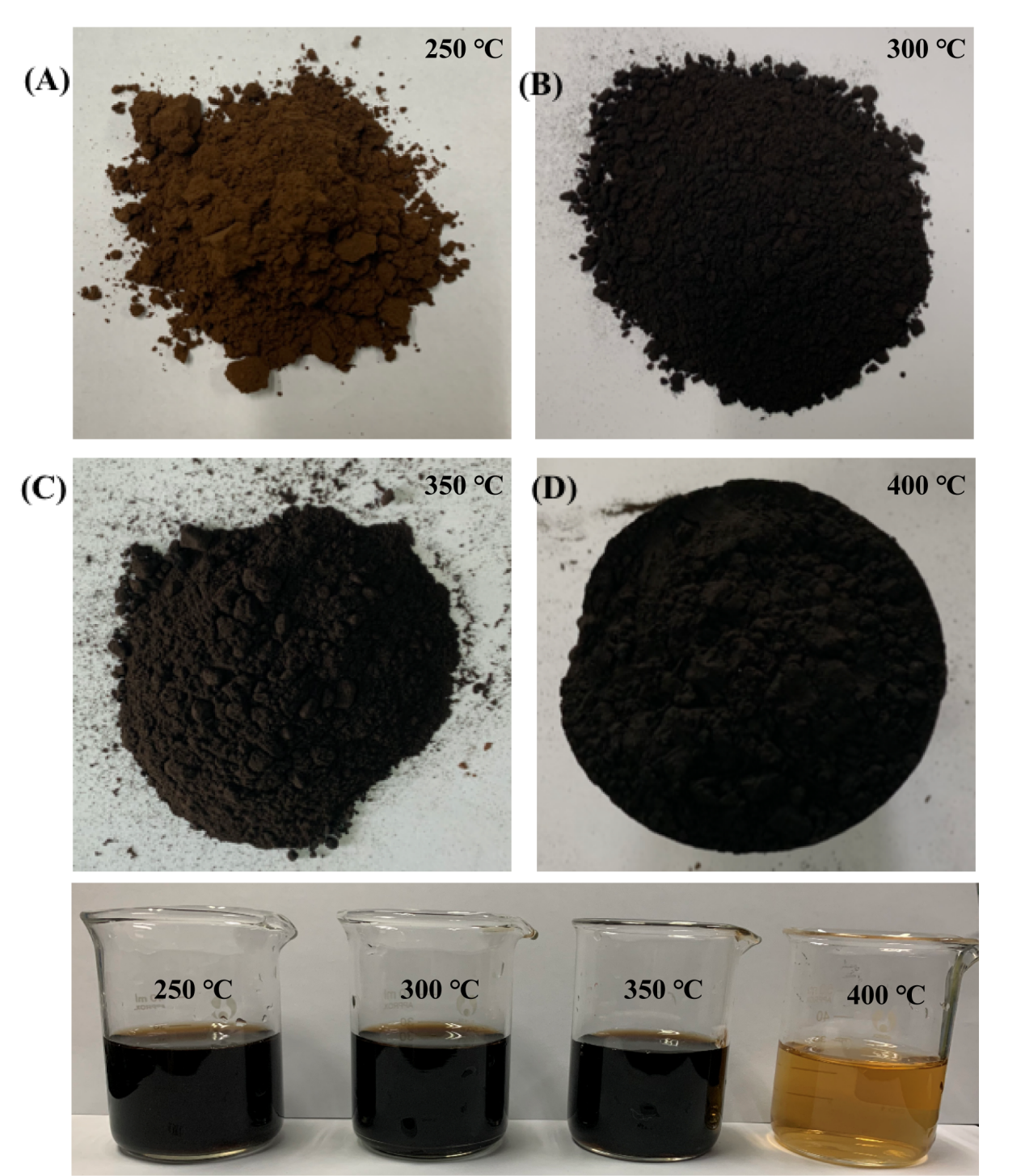


**Fig. S2** The appearance of PTC in different temperature (250 ℃, 300 ℃, 350 ℃ and 400 ℃).

**
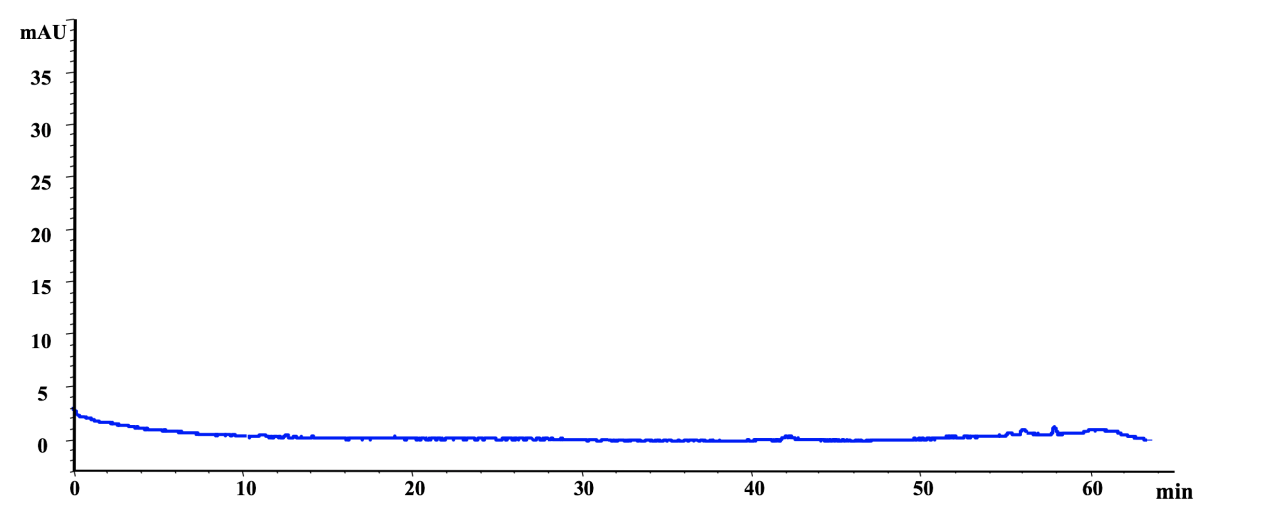
**

**Fig. S3** The image of HPLC assay on PT-CDs.

Reference:

1. Kong H, Zhao Y, Zhu Y, Xiong W, Luo J, Cheng J, Zhang Y, Zhang M, Qu H, Zhao Y: **Carbon dots from Artemisiae Argyi Folium Carbonisata: strengthening the anti-frostbite ability.** *Artif Cells Nanomed Biotechnol* 2021, **49:**11-19.

2. Zhao Y, Zhang Y, Kong H, Zhang M, Cheng J, Wu J, Qu H, Zhao Y: **Carbon Dots from Paeoniae Radix Alba Carbonisata: Hepatoprotective Effect.** *Int J Nanomedicine* 2020, **15:**9049-9059.

3. Lu F, Ma Y, Huang H, Zhang Y, Kong H, Zhao Y, Qu H, Wang Q, Liu Y, Kang Z: **Edible and highly biocompatible nanodots from natural plants for the treatment of stress gastric ulcers.** *Nanoscale* 2021, **13:**6809-6818.

4. Cheng JJ, Zhang ML, Sun ZW, Lu F, Xiong W, Luo J, Kong H, Wang QG, Qu HH, Zhao Y: **Hemostatic and hepatoprotective bioactivity of Junci Medulla Carbonisata-derived Carbon Dots.** *Nanomedicine* 2019, **14:**431-446.

5. Zhang ML, Cheng JJ, Zhang Y, Kong H, Wang SN, Luo J, Qu HH, Zhao Y: **Green synthesis of Zingiberis rhizoma-based carbon dots attenuates chemical and thermal stimulus pain in mice.** *Nanomedicine* 2020, **15:**851-869.

6. Zhang M, Cheng J, Hu J, Luo J, Zhang Y, Lu F, Kong H, Qu H, Zhao Y: **Green Phellodendri Chinensis Cortex-based carbon dots for ameliorating imiquimod-induced psoriasis-like inflammation in mice.** *J Nanobiotechnology* 2021, **19:**105.

7. Zhang ML, Cheng JJ, Sun ZW, Kong H, Zhang Y, Wang SN, Wang XK, Zhao Y, Qu HH: **Protective Effects of Carbon Dots Derived from Phellodendri Chinensis Cortex Carbonisata against Deinagkistrodon acutus Venom-Induced Acute Kidney Injury.** *Nanoscale Research Letters* 2019, **14:**13.

8. Liu XM, Wang YZ, Yan X, Zhang ML, Zhang Y, Cheng JJ, Lu F, Qu HH, Wang QG, Zhao Y: **Novel Phellodendri Cortex (Huang Bo)-derived carbon dots and their hemostatic effect.** *Nanomedicine* 2018, **13:**391-405.

9. Wu JS, Zhang ML, Cheng JJ, Zhang Y, Luo J, Liu YH, Kong H, Qu HH, Zhao Y: **Effect of Lonicerae japonicae Flos Carbonisata-Derived Carbon Dots on Rat Models of Fever and Hypothermia Induced by Lipopolysaccharide.** *International Journal of Nanomedicine* 2020, **15:**4139-4149.

10. Sun ZW, Lu F, Cheng JJ, Zhang ML, Zhang Y, Xiong W, Zhao Y, Qu HH: **Haemostatic bioactivity of novel Schizonepetae Spica Carbonisata-derived carbon dots via platelet counts elevation.** *Artificial Cells Nanomedicine and Biotechnology* 2018, **46:**S308-S317.

11. Yan X, Zhao Y, Luo J, Xiong W, Liu XM, Cheng JJ, Wang YZ, Zhang ML, Qu HH: **Hemostatic bioactivity of novel Pollen Typhae Carbonisata-derived carbon quantum dots.** *Journal of Nanobiotechnology* 2017, **15:**8.

12. Wang SN, Zhang Y, Kong H, Zhang ML, Cheng JJ, Wang XK, Lu F, Qu HH, Zhao Y: **Antihyperuricemic and anti-gouty arthritis activities of Aurantii fructus immaturus carbonisata-derived carbon dots.** *Nanomedicine* 2019, **14:**2925-2939.

13. Zhao Y, Zhang Y, Kong H, Cheng G, Qu H, Zhao Y: **Protective Effects of Carbon Dots Derived from Armeniacae Semen Amarum Carbonisata Against Acute Lung Injury Induced by Lipopolysaccharides in Rats.** *Int J Nanomedicine* 2022, **17:**1-14.

14. Xu Y, Wang B, Zhang M, Zhang J, Li Y, Jia P, Zhang H, Duan L, Li Y, Li Y, et al: **Carbon Dots as a Potential Therapeutic Agent for the Treatment of Cancer-Related Anemia.** *Adv Mater* 2022, **34:**e2200905.

15. Luo WK, Zhang LL, Li XX, Zheng J, Chen Q, Yang ZY, Cheng MH, Chen Y, Wu Y, Zhang W, et al: **Green functional carbon dots derived from herbal medicine ameliorate blood-brain barrier permeability following traumatic brain injury.** *Nano Research* 2022, **15:**9274-9285.

16. Gudimella KK, Gedda G, Kumar PS, Babu BK, Yamajala B, Rao BV, Singh PP, Kumar D, Sharma A: **Novel synthesis of fluorescent carbon dots from bio-based Carica Papaya Leaves: Optical and structural properties with antioxidant and anti-inflammatory activities.** *Environ Res* 2022, **204:**111854.
